# Specific persistent symptoms of COVID-19 and associations with reinfection: a community-based survey study in southern China

**DOI:** 10.3389/fpubh.2024.1452233

**Published:** 2024-09-03

**Authors:** Dongjing Liu, Binglin Chen, Xuejiao Liao, Zheng Zhang, Sen Wei, Xinxin Han, Yong Xu

**Affiliations:** ^1^The Third People’s Hospital of Shenzhen, Shenzhen, China; ^2^Shenzhen Research Center for Communicable Disease Diagnosis and Treatment, Chinese Academy of Medical Sciences, Shenzhen, China; ^3^School of Public Health and Emergency Management, Southern University of Science and Technology, Shenzhen, China; ^4^Longgang District Public Health Services Center, Shenzhen, China

**Keywords:** infectious disease, COVID-19, reinfection, persistent symptoms, community-based surveillance

## Abstract

**Background:**

Surveillance remains fundamental to understanding the changes in epidemiological patterns regarding post-COVID conditions and reinfections. Persistent symptoms and reinfection in previously infected individuals are increasing being reported in many countries, but their associations among general populations were seldomly reported. Understanding the association with persistent symptoms of COVID-19 reinfection is essential to develop strategies to mitigate the long-term health and socio-economic impacts of the post-COVID conditions. This study aimed to investigate the incidence of COVID-19 persistent symptoms among previously infected Chinese community residents and explore associations of specific COVID-19 persistent symptoms with reinfection and other factors.

**Methods:**

A community-based survey was conducted in a southern city of China with about 20 million residents from August 3 to 24, 2023. Face-to-face questionnaires were distributed to a total of 1,485 residents to collect their information about COVID-19 infection, reinfection, specific ongoing persistent symptoms, and other COVID-19 related information. Multivariable logistic regression analysis was used to examine the association between specific persistent symptoms and reinfection of COVID-19, along with age, gender, and educational level.

**Results:**

Of the 1,485 participants, 1,089 (73.3%) reported they had been infected with COVID-19. Among them, 89.1% reported having ongoing persistent symptoms and 14.2% reported had two or more times of infection. About 20% participants were infected 1 year or more since their initial infection. Fatigue, cough, and headaches were the top 3 symptoms being reported. Participants with reinfection were associated with a higher probability of reporting headaches (OR: 1.54, 95% CI: 1.06–2.25), loss of or change in smell and/or taste (OR: 1.90, 95% CI: 1.27–2.83), impaired sleep (OR: 1.55, 95% CI: 1.02–2.35), and brain fog (OR: 1.76, 95% CI: 1.12–2.76). Participants aged 45 and above and who had a bachelor’s or higher degree were more likely to report chest tightness or shortness of breath, impaired sleep, and brain fog.

**Discussion:**

During the post-emergency period of COVID-19 pandemic, the incidence of ongoing persistent symptoms among Chinese residents remains high. Individuals whose initial infection was longer than 1 year have the highest probability of reporting having multiple symptoms. Reinfection may increase the risk of reporting headaches, loss of or change in smell and/or taste, impaired sleep, and brain fog. It is important to maintain routine syndromic surveillance among previously infected people and provide recommendations for clinical management of individuals with multiple ongoing symptoms.

## 1 Introduction

Surveillance remains fundamental to understanding the changes in epidemiological patterns regarding post-COVID conditions and reinfections. Post-COVID-19 symptoms have been increasingly reported among individuals previously infected with SARS-CoV-2 in many countries (1–4). The Institute for Health Metrics and Evaluation estimated that by the end of 2021, globally, 3.7% (144.7 million) of 3.92 billion individuals who had been infected with SARS-CoV-2 developed post COVID-19 condition, with 15.1% (22 million) having persistent symptoms at 12 months after infection onset (5). A more recent study estimated that about 10% of over 651 million infected individuals experienced persistent symptoms globally, and this number is still rising (6). The commonly reported persistent symptoms following SARS-CoV-2 infection include fatigue, general pain or discomfort, impaired sleep, breathlessness, and impaired usual activity (2).

The incidence of reinfection is also commonly reported among people previously infected with SARS-CoV-2. A systematic review indicated that the SARS-CoV-2 reinfection incidence rate was 0.70 per 10,000 person-days and that the incidence of reinfection was lower than the incidence of new infection (7). Another systematic review estimated that the number of reinfections among patients who recovered from COVID-19 was 3 per 1,000 patients (8). Cough and fever were reported as the main symptoms of both the first infection and reinfection (9). A Chinese survey study reported a 12.6% reinfection incidence among the 999 respondents and a higher reinfection incidence of females than that of males (10).

Persistent symptoms after COVID-19 have a significant impact on patients’ long-term health and quality of life (11). Thus far, evidence about persistent symptoms based on population surveys mostly comes from the United States and European countries such as Netherlands, England; evidence from Chinese residents at the community level is relatively less reported. A prior study in Wuhan in 2021 surveyed 1,791 community residents who were COVID-19 survivors, but this study only reported the phenotype clusters of long-term sequelae of COVID-19 without detail conditions (4). In addition, research that explore the associations of persistent symptoms with reinfection is also limited. A prior US study that used Veterans’ health administrative data demonstrated that reinfection may increase the risk of having persistent symptoms (3), but it did not investigate the association of reinfections with detailed symptoms.

In this study, we aimed to investigate patterns of COVID-19 persistent symptoms among Chinese community residents, as well as to explore associations of specific COVID-19 persistent symptoms with reinfection. This community-based surveillance study provides the most recent evidence for changes in epidemiological patterns regarding post-COVID conditions and reinfections among Chinese general residents, which can help to inform public health actions to further limiting the long-term impact of this global pandemic.

## 2 Materials and methods

### 2.1 Design, setting, and participants

A community-based survey was conducted in Shenzhen, a southern city of China with about 20 million residents, from August 3 to 24, 2023. The duration of the recall period were 22 days. Seven communities in the city were randomly selected as the study sites, according to the economic level and geographic location. A face-to-face survey was distributed by trained epidemiological investigators to residents to collect their information about COVID-19 infection, reinfection, specific ongoing persistent symptoms, and residents’ basic demographic characteristics (e.g., age, gender, and educational level) (12, 13). Participants were enrolled by stratified random sampling according to gender and age. Thus, the sample was representativeness of Shenzhen residents. The questionnaire was filled out with the help of trained investigators due to factors such as respondents’ vision. A pilot study was conducted among 219 residents to ensure that the questionnaire was easily understood and completed before the questionnaire was sent to all participants. The surveyed individuals were all voluntary participated and obtained oral informed consents before filling in the questionnaire.

The study was approved by the Institutional Review Board of the Third People’s Hospital of Shenzhen.

### 2.2 Measurements

(1) For reinfection, the survey asked participants to report the number of times they had been infected with COVID-19. We created a dummy variable for individuals who reported being infected twice or more with COVID-19 to indicate COVID-19 reinfection. We also calculated interval from initial infection to investigation for each respondent.

We reported the methods that infected individuals used for confirming their infection either through nucleic acid test, antigen detection, or self-speculation.

(2) For persistent symptoms, the survey asked participants to report if they had any of the following symptoms after initial COVID-19 infection: fatigue, cough, chest tightness or shortness of breath, chest pain, dizziness, headaches, brain fog (such as problems of concentrating or thinking), loss of or change in smell and/or taste, diarrhea, hair loss, joint pain, muscle pain, impaired sleep, and changes in emotions (such as having depression or anxiety). We created dummy variables for individuals who reported a particular symptom and calculated the number of symptoms they reported.

### 2.3 Statistical analysis

Data were entered and managed using EpiData 3.1 and analyzed using Stata version 17.0 (StataCorp Inc., Chicago, USA). Categorical variables were compared using Chi-squared tests. A multivariable logistic regression analysis was performed to examine the association between reinfection and the top-9 rated reported symptoms, along with participants’ age, gender, educational level and other factors. Odds ratio (OR) and the corresponding 95% confidence interval (CI) were reported. A *p*-value less than 0.05, with two-tail tests, was considered statistically significant. All analyses were performed using Stata version 17.0.

## 3 Results

### 3.1 Basic characteristics of respondents

A total of 1,485 participants completed the survey and 1,089 (73.3%) reported previously infected with SARS-CoV-2. Of the 1,485 participants, 57.8% were female, 19.2% were aged 45 and above, and 47.7% had a bachelor’s or higher degree, (Table 1).

**Table 1:** Basic demographics of 1,485 participants in southern China.

	Frequency	% (95% CI)
Gender		
Male	627	42.2 (39.7–44.8)
Female	858	57.8 (55.2–60.3)
Age		
less than 30	481	32.4 (30.1–34.8)
30–44	718	48.4 (45.8–50.9)
45 and above	286	19.2 (17.3–21.3)
Educational level		
Junior high school and below	405	27.3 (25.1–29.6)
Senior high school/Technical secondary school	371	25.0 (22.8–27.3)
University/College and above	709	47.7 (45.3–50.3)
Infected status		
None	396	26.7 (24.5–29.0)
Infected once	935	63.0 (60.5–65.4)
Reinfection (infected twice or more)	154	10.3 (8.9–12.0)

### 3.2 Reinfected status and ongoing symptoms of previously infected respondents

Of these 1,089 previously infected individuals, 14.1% reported being infected twice or more with COVID-19, and 71.0% were initially infected between 6 months to 1 year before they were surveyed (Table 2). About 984 (90.4%) reported having ongoing persistent symptoms after initial infection and 70.3% reported having at least two ongoing persistent symptoms. The top-9 rated symptoms reported by these respondents were fatigue (46.7%), cough (44.3%), headaches (23.5%), dizziness (19.7%), chest tightness or shortness of breath (18.2%), muscle pain (18.0%), loss of or change in smell and/or taste (17.9%), sleepless problems (17.1%), and brain fog (13.3%).

**Table 2:** Reinfection status and persistent symptoms of COVID-19 in 1,089 previously infected participants.

	Frequency	% (95% CI)
Reinfection status		
Infected once	935	85.9 (83.7–87.8)
Reinfected (infected twice or more)	154	14.1 (12.2–16.3)
Interval from initial infection to investigation		
< 6 months	90	8.3 (6.8–10.1)
6 months to 1 year	773	71.0 (68.2–73.6)
> 1 year	226	20.8 (18.4–23.3)
Whether having ongoing persistent symptoms		
No	105	9.6 (8.0–11.5)
Yes	984	90.4 (88.5–92.0)
Number of persistent symptoms reported		
One symptom	292	29.7 (26.9–32.6)
Two or more symptoms	692	70.3 (67.4–73.1)
Specific persistent symptoms reported		
Fatigue	516	47.4 (44.4–50.4)
Cough	489	44.9 (41.9–47.9)
Headaches	256	23.5 (21.0–26.1)
Dizziness	218	20.0 (17.7–22.5)
Chest tightness or shortness of breath	198	18.2 (15.9–20.6)
Muscle pain	198	18.2 (15.9–20.6)
Loss of or change in smell and/or taste	196	18.0 (15.8–20.4)
Impaired sleep	188	17.3 (15.1–19.7)
Brain fog (such as problems of concentrating or thinking)	146	13.4 (11.4–15.6)
Joint pain	131	12.0 (10.2–14.1)
Chest pain	127	11.7 (9.8–13.7)
Changes in emotions (such as having depression or anxiety)	76	7.0 (5.5–8.7)
Hair loss	70	6.4 (5.0–8.1)
Diarrhea	63	5.8 (4.5–7.3)

### 3.3 Incidence of ongoing persistent symptoms by reinfection status, interval from initial infection to investigation, confirmation of infection, and basic characteristics of respondents

The results showed that individuals with reinfection was significantly more likely to report having two or more ongoing symptoms than that of non-reinfected individuals (83.1% vs. 60.3%, *p* < 0.001) (Table 3). The earlier the initial infection occurred, the higher the probability of having two or more ongoing symptoms (53.3% for those initially infected less than 6 months, 62.1% for those initially infected between 6 months to 1 year, and 72.6% for those initially infected more than 1 year, *p* = 0.003). Individuals with infections confirmed through nucleic acid tests were significantly more likely to report two or more ongoing symptoms than individuals using other testing methods (67.3% for nucleic acid tests, 60.5% for antigen detection, and 65.7% for self-speculation, *p* = 0.031). Incidence of having two or more symptoms was also varied by different age groups (58.3% for less than 30, 66.2% for 30–44, and 65.1% for 45 and above, *p* = 0.004).

**Table 3:** Incidence of ongoing persistent symptoms by reinfection status, interval from initial infection to investigation, confirmation of infection, and basic demographic characteristics of 1,089 participants.

	Number of persistent symptoms			*χ*2	*p*-value
	None (*N* = 105)	One symptom (*N* = 292)	Two or more symptoms (*N* = 692)		
Reinfection status, *n (%)*				31.474	<0.001
Infected once	94 (10.1)	277 (29.6)	564 (60.3)		
Reinfected	11 (7.2)	15 (9.7)	128 (83.1)		
Interval from initial infection to investigation, *n (%)*				15.698	0.003
< 6 months	8 (8.9)	34 (37.8)	48 (53.3)		
6 months to 1 year	84 (10.9)	209 (27.0)	480 (62.1)		
> 1 year	13 (5.7)	49 (21.7)	164 (72.6)		
Confirmation of infection, *n (%)*				10.664	0.031
Self-speculation	22 (13.0)	36 (21.3)	111 (65.7)		
Antigen detection	50 (8.9)	173 (30.6)	342 (60.5)		
Nucleic acid test (NAT)	33 (9.3)	83 (23.4)	239 (67.3)		
Gender, *n (%)*				0.829	0.661
Male	41 (9.0)	118 (25.9)	296 (65.1)		
Female	64 (10.1)	174 (27.4)	396 (62.5)		
Age, *n (%)*				15.298	0.004
less than 30	27 (7.9)	116 (33.8)	200 (58.3)		
30–44	62 (11.3)	124 (22.5)	365 (66.2)		
45 and above	16 (8.2)	52 (26.7)	127 (65.1)		
Educational level, *n (%)*				1.976	0.740
Junior high school and below	29 (11.3)	66 (25.7)	162 (63.0)		
Senior high school/Technical secondary school	21 (8.3)	65 (25.7)	167 (66.0)		
University/College and above	55 (9.5)	161 (27.8)	363 (62.7)		

### 3.4 Adjusted associations of the top-9 ranked persistent symptoms with reinfection and other characteristics

**Figure 1:**
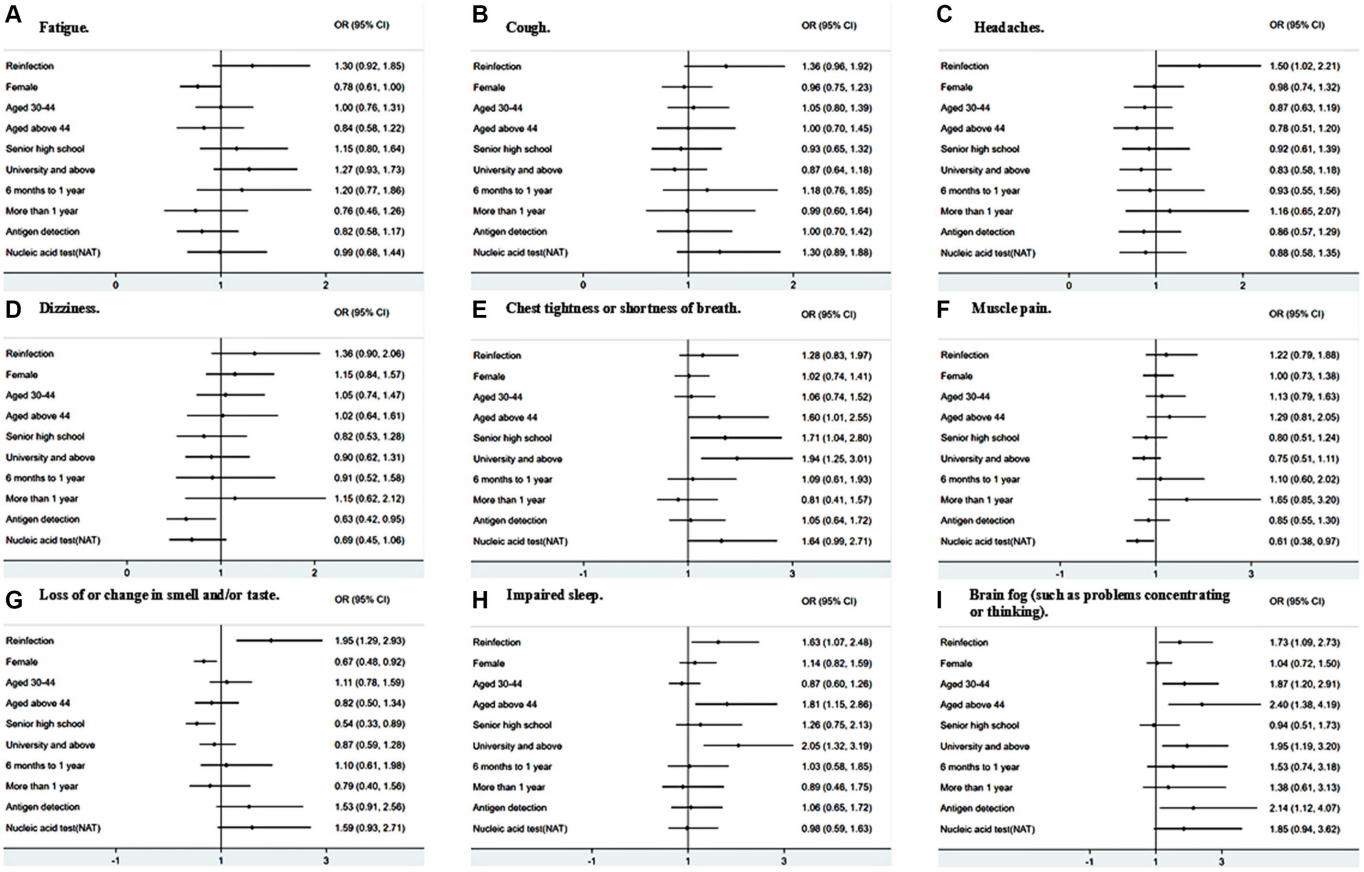
Adjusted associations of top-9 ranked persistent symptoms with reinfection and other factors.

The multivariable logistic regression analysis showed that, compared to participants who were infected only once with COVID-19, participants who had been reinfected were more likely to report headaches (OR: 1.54, 95% CI: 1.06–2.25, *p* = 0.024), loss of or change in smell and/or taste (OR: 1.90, 95% CI: 1.27–2.83, *p* = 0.002), sleepless problems (OR: 1.55, 95% CI: 1.02–2.35, *p* = 0.038), and brain fog (OR: 1.76, 95% CI: 1.12–2.76, *p* = 0.014) (Figure 1). Female participants, compared to male participants were less likely to report loss of or change in smell (OR: 0.67, 95% CI: 0.49–0.92, *p* = 0.013). Compared to participants aged below 30, participants aged 45 and above were more likely to report chest tightness or shortness of breath (OR: 1.61, 95% CI: 1.01–2.54, *p* = 0.043), impaired sleep (OR: 1.73, 95% CI: 1.09–2.73, *p* = 0.020), and brain fog (OR: 2.44, 95% CI: 1.40–4.25, *p* = 0.002). Compared to participants who had junior high school and below education, participants who had a bachelor’s or higher degree were more likely to report chest tightness or shortness of breath (OR: 1.90, 95% CI: 1.23–2.93, *p* = 0.004), impaired sleep (OR: 2.02, 95% CI: 1.30–3.12, *p* = 0.002), and brain fog (OR: 2.18, 95% CI: 1.33–3.58, *p* = 0.002).

## 4 Discussion

This study was conducted during August 2023, 8 months after the massive outbreak of infection in December 2022, when China lessened its containment policy. In this study of 1,089 community residents who were previously infected with SARS-CoV-2 in southern China, we investigated patterns of COVID-19 persistent symptoms of among Chinese community residents who were previously infected with SARS-CoV-2, as well as to explore the associations of specific COVID-19 persistent symptoms with reinfection.

We found that 89.1% of previously infected individuals reported having ongoing persistent symptoms, and 70.3% reported having at least two ongoing persistent symptoms. The most commonly reported symptoms were fatigue, cough, headaches, dizziness, and chest tightness or shortness of breath. The high rate of reporting ongoing symptoms overall and, in particular, symptoms, such as fatigue, headaches, and brain fog, were consistent with prior studies in other countries (14). We also found a 12.6% reinfection incidence among previously infected individuals, which the results are consistent with previous studies that investigated in eastern China (10).

Our study revealed differences in some reported symptoms between individuals with reinfection and initial infection and by different demographics. We added to the literature that individuals whose initial infection longer than 1 year have the highest probability of reporting having multiple symptoms. We did not investigate the specific strains, but we suspected possible strains based on the timing respondents reported being infected. Respondents who reported being infected during 2020 could be highly likely to be infected with the original strain, respondents who reported being infected after May 2021 could be highly likely to be infected with the Delta strain. For those who reported being infected after November 2021, they might be highly likely to be infected with the Omicron strain. Being infected more times was also associated with a higher probability of reporting persisting symptoms, such as headaches, loss of or change in smell, impaired sleep, and brain fog. These findings were consistent with the US study that found that those with reinfections had an increased risk of at least one persistent symptom than those being initially infected (15). Our study was more detailed by presenting the association of one particular symptom with reinfection. Additionally, we found that higher age and higher educational level were associated with a higher probability of reporting chest tightness or shortness of breath, impaired sleep, and brain fog, which has not been reported elsewhere.

We acknowledged several limitations of this study. First, there was some reporting and recall bias. For example, there were differences in how individuals perceive and report symptoms; some may not report them because they are mild. In addition, as the recall period increased, participants may perceive recent symptoms as persistent symptoms, and this memory inaccuracy may affect the reporting of persistent symptoms. Since self-reported symptoms are highly dependent on an individual’s perception and memory, this bias can be significant and affect the assessment of the prevalence of persistent symptoms. Second, we did not compare the data to COVID-19 negative controls, but prior studies that used non-COVID-19 controls found a relatively lower prevalence of similar symptoms. Third, since the data was self-reported, we lack clinical diagnosis for infections, although we asked participants the way they confirmed their infection, and more than 85% of participants confirmed infection through nucleic acid testing or antigen detection. Fourth, due to the observational nature of study design, our study can only detect an association but cannot reveal a causal relationship between COVID-19 reinfection and persistent symptoms. It is likely that other diseases unmeasured in this study also induced the symptoms we investigated. Lastly, we only included basic demographic characteristics of respondents (i.e., age, gender, and educational level) in the analysis. Failing to include other social and epidemiological characteristics such as income, occupation, and chronic disease history, might influence the results. Future research should focus more on the potential impact of sociodemographic factors.

## 5 Conclusion

This community-based survey reported data about the incidence of ongoing persistent symptoms and reinfection among 1,089 residents previously infected with SARS-CoV-2 during the post-emergency period of COVID-19 pandemic. Prolonged symptoms occurred in many residents after initial infection, and those whose initial infection was longer than 1 year have the highest probability of reporting having multiple symptoms. Reinfection appears to increase the risk of reporting headaches, loss of or change in smell and/or taste, impaired sleep, and brain fog. It is important to maintain routine syndromic surveillance among previously infected people and provide recommendations for clinical management of individuals with multiple ongoing symptoms.

## Data Availability

The raw data supporting the conclusions of this article will be made available by the authors, without undue reservation.
