# Supplementary material for: Specific persistent symptoms of COVID-19 and associations with reinfection: a community-based survey study in southern China
Source: Front Public Health. 2024 Sep 3;12:1452233. doi: 10.3389/fpubh.2024.1452233 (PMC11405231; doi:10.3389/fpubh.2024.1452233)
Supplement: Supplementary file 1 [file Table_1.docx]

Appendix 1. Multivariate logistic regression of the top-9 persistent symptoms of COVID-19.

| **items** | **Fatigue** | | **Cough** | | **Headaches** | | **Dizziness** | | **Chest tightness or shortness of breath** | |
| --- | --- | --- | --- | --- | --- | --- | --- | --- | --- | --- |
|  | **OR (95%CI)** | ***P-value*** | **OR (95%CI)** | ***P-value*** | **OR (95%CI)** | ***P-value*** | **OR (95%CI)** | ***P-value*** | **OR (95%CI)** | ***P-value*** |
| **Repeated COVID-19 infection** |  |  |  |  |  |  |  |  |  |  |
| Once infection |  |  |  |  |  |  |  |  |  |  |
| Repeated COVID-19 infection / Reinfection | 1.30 (0.92-1.85) | 0.140 | 1.36 (0.96-1.92) | 0.084 | 1.50 (1.02-2.21) | 0.037 | 1.36 (0.90-2.06) | 0.138 | 1.28 (0.83-1.97) | 0.269 |
| **Gender** |  |  |  |  |  |  |  |  |  |  |
| Male |  |  |  |  |  |  |  |  |  |  |
| Female | 0.78 (0.61-1.00) | 0.049 | 0.96 (0.75-1.23) | 0.769 | 0.98 (0.74-1.32) | 0.911 | 1.15 (0.84-1.57) | 0.376 | 1.02 (0.74-1.41) | 0.905 |
| **Age** |  |  |  |  |  |  |  |  |  |  |
| less than 30 |  |  |  |  |  |  |  |  |  |  |
| 30-44 | 1.00 (0.76-1.31) | 0.993 | 1.05 (0.80-1.39) | 0.702 | 0.87 (0.63-1.19) | 0.384 | 1.05 (0.74-1.47) | 0.793 | 1.06 (0.74-1.52) | 0.751 |
| 45 and above | 0.84 (0.58-1.22) | 0.367 | 1.00 (0.70-1.45) | 0.980 | 0.78 (0.51-1.20) | 0.264 | 1.02 (0.64-1.61) | 0.938 | 1.60 (1.01-2.55) | 0.046 |
| **Educational level** |  |  |  |  |  |  |  |  |  |  |
| Junior high school and below |  |  |  |  |  |  |  |  |  |  |
| Senior high school / Technical secondary school | 1.15 (0.80-1.64) | 0.452 | 0.93 (0.65-1.32) | 0.677 | 0.92 (0.61-1.39) | 0.690 | 0.82 (0.53-1.28) | 0.389 | 1.71 (1.04-2.80) | 0.034 |
| University / College and above | 1.27 (0.93-1.73) | 0.128 | 0.87 (0.64-1.18) | 0.359 | 0.83 (0.58-1.18) | 0.302 | 0.90 (0.62-1.31) | 0.585 | 1.94 (1.25-3.01) | 0.003 |
| **Interval from initial infection to investigation** |  |  |  |  |  |  |  |  |  |  |
| < 6 months |  |  |  |  |  |  |  |  |  |  |
| 6 months to 1 year | 1.20 (0.77-1.86) | 0.428 | 1.18 (0.76-1.85) | 0.459 | 0.93 (0.55-1.56) | 0.774 | 0.91 (0.52-1.58) | 0.734 | 1.09 (0.61-1.93) | 0.779 |
| > 1 year | 0.76 (0.46-1.26) | 0.291 | 0.99 (0.60-1.64) | 0.964 | 1.16 (0.65-2.07) | 0.626 | 1.15 (0.62-2.12) | 0.665 | 0.81 (0.41-1.57) | 0.529 |
| **Way of diagnosis** |  |  |  |  |  |  |  |  |  |  |
| Self-speculation |  |  |  |  |  |  |  |  |  |  |
| Antigen detection | 0.82 (0.58-1.17) | 0.280 | 1.00 (0.70-1.42) | 0.989 | 0.86 (0.57-1.29) | 0.467 | 0.63 (0.42-0.95) | 0.028 | 1.05 (0.64-1.72) | 0.849 |
| Nucleic acid test (NAT) | 0.99 (0.68-1.44) | 0.971 | 1.30 (0.89-1.88) | 0.173 | 0.88 (0.58-1.35) | 0.572 | 0.69 (0.45-1.06) | 0.093 | 1.64 (0.99-2.71) | 0.056 |

Appendix 1. Multivariate logistic regression of the top-9 persistent symptoms of COVID-19 (continued).

| **items** | **Muscle pain** | | **Loss of or change in smell and/or taste** | | **Impaired sleep** | | **Brain fog (such as problems concentrating or thinking)** | |
| --- | --- | --- | --- | --- | --- | --- | --- | --- |
|  | **OR (95%CI)** | ***P-value*** | **OR (95%CI)** | ***P-value*** | **OR (95%CI)** | ***P-value*** | **OR (95%CI)** | ***P-value*** |
| **Repeated COVID-19 infection** |  |  |  |  |  |  |  |  |
| Once infection |  |  |  |  |  |  |  |  |
| Repeated COVID-19 infection / Reinfection | 1.22 (0.79-1.88) | 0.361 | 1.95 (1.29-2.93) | 0.001 | 1.63 (1.07-2.48) | 0.023 | 1.73 (1.09-2.73) | 0.019 |
| **Gender** |  |  |  |  |  |  |  |  |
| Male |  |  |  |  |  |  |  |  |
| Female | 1.00 (0.73-1.38) | 0.976 | 0.67 (0.48-0.92) | 0.013 | 1.14 (0.82-1.59) | 0.423 | 1.04 (0.72-1.50) | 0.847 |
| **Age** |  |  |  |  |  |  |  |  |
| less than 30 |  |  |  |  |  |  |  |  |
| 30-44 | 1.13 (0.79-1.63) | 0.494 | 1.11 (0.78-1.59) | 0.558 | 0.87 (0.60-1.26) | 0.468 | 1.87 (1.20-2.91) | 0.006 |
| 45 and above | 1.29 (0.81-2.05) | 0.286 | 0.82 (0.50-1.34) | 0.432 | 1.81 (1.15-2.86) | 0.011 | 2.40 (1.38-4.19) | 0.002 |
| **Educational level** |  |  |  |  |  |  |  |  |
| Junior high school and below |  |  |  |  |  |  |  |  |
| Senior high school / Technical secondary school | 0.80 (0.51-1.24) | 0.318 | 0.54 (0.33-0.89) | 0.016 | 1.26 (0.75-2.13) | 0.376 | 0.94 (0.51-1.73) | 0.848 |
| University / College and above | 0.75 (0.51-1.11) | 0.147 | 0.87 (0.59-1.28) | 0.480 | 2.05 (1.32-3.19) | 0.001 | 1.95 (1.19-3.20) | 0.008 |
| **Interval from initial infection to investigation** |  |  |  |  |  |  |  |  |
| < 6 months |  |  |  |  |  |  |  |  |
| 6 months to 1 year | 1.10 (0.60-2.02) | 0.749 | 1.10 (0.61-1.98) | 0.760 | 1.03 (0.58-1.85) | 0.912 | 1.53 (0.74-3.18) | 0.252 |
| > 1 year | 1.65 (0.85-3.20) | 0.138 | 0.79 (0.40-1.56) | 0.497 | 0.89 (0.46-1.75) | 0.744 | 1.38 (0.61-3.13) | 0.444 |
| **Way of diagnosis** |  |  |  |  |  |  |  |  |
| Self-speculation |  |  |  |  |  |  |  |  |
| Antigen detection | 0.85 (0.55-1.30) | 0.452 | 1.53 (0.91-2.56) | 0.107 | 1.06 (0.65-1.72) | 0.822 | 2.14 (1.12-4.07) | 0.021 |
| Nucleic acid test (NAT) | 0.61 (0.38-0.97) | 0.036 | 1.59 (0.93-2.71) | 0.091 | 0.98 (0.59-1.63) | 0.935 | 1.85 (0.94-3.62) | 0.074 |
